# Immunisation Information Systems – useful tools for monitoring vaccination programmes in EU/EEA countries, 2016

**DOI:** 10.2807/1560-7917.ES.2017.22.17.30519

**Published:** 2017-04-27

**Authors:** Tarik Derrough, Kate Olsson, Vincenza Gianfredi, Francois Simondon, Harald Heijbel, Niklas Danielsson, Piotr Kramarz, Lucia Pastore-Celentano

**Affiliations:** 1 European Centre for Disease Prevention and Control (ECDC), Stockholm, Sweden; 2 School of Specialization in Hygiene and Preventive Medicine, Department of Experimental Medicine, University of Perugia, Perugia, Italy; 3 Mother and Child Health research unit 216, IRD & Paris Descartes University, France; 4 Ret. Swedish Institute for Infectious Disease Control (SMI), Stockholm, Sweden.

**Keywords:** vaccines, immunisation, immunisation information systems, vaccination coverage, European Union

## Abstract

Immunisation Information Systems (IIS) are computerised confidential population based-systems containing individual-level information on vaccines received in a given area. They benefit individuals directly by ensuring vaccination according to the schedule and they provide information to vaccine providers and public health authorities responsible for the delivery and monitoring of an immunisation programme. In 2016, the European Centre for Disease Prevention and Control (ECDC) conducted a survey on the level of implementation and functionalities of IIS in 30 European Union/European Economic Area (EU/EEA) countries. It explored the governance and financial support for the systems, IIS software, system characteristics in terms of population, identification of immunisation recipients, vaccinations received, and integration with other health record systems, the use of the systems for surveillance and programme management as well as the challenges involved with implementation. The survey was answered by 27 of the 30 EU/EEA countries having either a system in production at national or subnational levels (n = 16), or being piloted (n = 5) or with plans for setting up a system in the future (n = 6). The results demonstrate the added-value of IIS in a number of areas of vaccination programme monitoring such as monitoring vaccine coverage at local geographical levels, linking individual immunisation history with health outcome data for safety investigations, monitoring vaccine effectiveness and failures and as an educational tool for both vaccine providers and vaccine recipients. IIS represent a significant way forward for life-long vaccination programme monitoring.

## Introduction

Immunisation Information Systems (IIS) are defined as confidential, population-based, computerised databases that record all immunisation doses administered by participating providers to persons residing within a given geopolitical area [1]. At the point of clinical care, they support practitioner decision-making in ensuring appropriate individual vaccination and adherence to applicable policies. At population level, IIS provide aggregate data on vaccinations for use in surveillance and programme operations, and in guiding public health action with the goals of improving vaccination rates and reducing vaccine-preventable disease.

Following the introduction of a vaccine, its uptake and benefit-risk profile requires continuous assessment in order to monitor the performance of vaccination programmes [2,3] and to respond to national and international public health monitoring requirements (e.g. reporting on vaccination coverage, responding to post-licensure requirements, investigation of safety signals). One of the key performance indicators of a well-functioning immunisation programme is vaccination coverage – the proportion of the population eligible for vaccination that has been immunised. It is an indirect measurement of population immunity and determines the level of herd protection against vaccine preventable diseases. Historically, coverage assessment in European Union (EU) Member States has been performed through regular surveys (e.g. telephone-based, at school-entry), review of claims and social security databases or analysis of data from paper-based registries [4-10]. IIS can be a key tool for monitoring vaccination coverage. They can also facilitate evaluation of the safety and effectiveness of vaccines through linking individual vaccination data with other records on health outcomes [11-14]. The functionalities of such systems, including electronic patient records in the framework of e-Health initiatives, are developing rapidly and they should be able to provide useful information to public health authorities, vaccine providers and vaccine recipients.

For an IIS to fully support vaccination programmes, there are various features that are considered important. These can include: (i) complete and accurate denominator populations from different sources; (ii) secure vaccine recipient and record identification through uniform unique identifiers (UID); (iii) complete, timely and correct vaccination records with real-time electronic access to the IIS; (iv) recording of vaccinations given to the recipient and vaccine details (batch and vial ID etc.) facilitated by pre-entered information, selection menus and reading of barcodes; (v) production of automated outputs; (vi) the facility to offer services that are useful to all parties including vaccine recipients, parents and vaccine providers. This includes for example: recall functions, trusted medical information, and the possibility for parents and vaccine recipients to request certified records of immunisation history.

The European Council conclusions on childhood immunisation in 2011 and on vaccinations as an effective tool in public health in 2014 both recommend the adoption of such systems and the World Health Organization European Vaccine Action Plan 2015–2020 recognises IIS as ‘an integral part of well-functioning health systems’ [15-17].

This article presents the findings of a survey conducted by the European Centre for Disease Prevention and Control (ECDC) across EU/European Economic Area (EEA) countries that assessed the level of implementation of IIS and their functionalities, as well as the challenges encountered during the design and implementation. The aim of the survey was to share knowledge about IIS in the EU/EEA in order to build consensus on the characteristics of an optimal system and to describe differences in core functionalities and standards across countries.

## Methods

Following a review of the literature and in consultation with subject-matter experts, two cross-sectional surveys were developed to assess the status of IIS implementation and functionalities in EU/EEA countries [18,19].

The first, more comprehensive survey, which included 100 questions, targeted countries with an IIS in operation or being piloted. The other, briefer survey (including nine questions), targeted countries with no IIS or IIS at a very early stage of implementation. The surveys can be found on the ECDC website [20]. Respondents decided on the survey they would like to answer based on their national or subnational situation regarding IIS implementation status.

The full comprehensive survey explored the current status of IIS implementation, governance, regulation and financial sustainability, population covered, nature of the data recorded, technical solutions used, linkage with other health information systems, outputs generated, and challenges and barriers to implementation.

The briefer survey examined the current status of IIS implementation, barriers to the planning or implementing of IIS, plans for the future, and if there was a strategy for e-Health in place.

The surveys opened on 1 May 2016 and closed on 20 May 2016. Countries that could not complete either of the two surveys by the deadline were asked to complete a basic set of five questions.

In May 2016, the 28 EU Member States plus two EEA countries (Norway and Iceland) were invited to participate in the surveys. Respondents were identified through the ECDC National Focal Points (NFPs) for Vaccine Preventable Diseases (VPD).

The EU survey tool was used to administer the survey [21]. In countries with more than one system, the survey was limited to the IIS that covered the largest population. All survey data were analysed in Excel. 

The United States Centers for Disease Prevention and Control (US CDC) definition of an IIS was used as a reference in this survey [1] (Box).

Box **United States Centers for Disease Control and Prevention (US CDC) Immunisation Information Systems (IIS) definition [1]**

IIS are confidential, population-based, computerized databases that record all immunisation doses administered by participating providers to persons residing within a given geopolitical area.

At the point of clinical care, an IIS can provide consolidated immunisation histories for use by a vaccination provider in determining appropriate client vaccinations.

At the population level, an IIS provides aggregate data on vaccinations for use in surveillance and programme operations, and in guiding public health action with the goals of improving vaccination rates and reducing vaccine-preventable disease.

## Results

### Participation in the different surveys

Information was received from 27 countries of the 30 contacted, with 16 countries answering the full comprehensive survey, nine countries answering the brief survey and two countries (Luxembourg and Slovakia) answering only to the basic set of five questions.

The list of responding institutions and which survey they completed is shown in Table 1. The respondents were staff from public institutions at national or subnational level with responsibility for national vaccination programme or IIS managers.

**Table 1:** Institutions in EU/EEA countries that participated in ECDC surveys on IIS implementation, 2016 (n = 27 countries/institutions)

**Countries with respective institutions responding to the comprehensive survey (n = 16)**	
Belgium	Ministry of Social Affairs, Pubic Health and Environment, Scientific Institute for Public Health
Denmark	Statens Serum Institut, Department of Epidemiology Research
Finland	National Institute for Health and Welfare, Department of Vaccination and Immune Protection
Germany	Robert Koch Institute, Infectious Disease Epidemiology
Hungary	National Center for Epidemiology, Department of Communicable Diseases Epidemiology
Iceland	Centre for Health Security and Communicable Disease Control, Directorate of Health
Ireland	National Immunisation Office, National Immunisation and Child health Information System
Latvia	Centre for Disease Prevention and Control, Infectious Diseases Risk Analysis and Prevention Department
Malta	Ministry for Health, Department for Health Regulation – Health Promotion and Disease Prevention
Netherlands	National Institute for Public Health and the Environment, Centre for Infectious Disease Control
Norway	Public Health Institute, Norwegian Immunisation Registry
Portugal	Department of Disease prevention and Health Promotion, Directorate General for Health (DGS)
Romania	National Institute of Public Health, National Centre for Communicable Diseases Surveillance and Control
Spain	Ministry of health, Social Services and Equality, Immunization Programme
Sweden	Public Health Agency, Unit for Vaccination Programs
United Kingdom	Public Health England, Department of Immunisation, Hepatitis and Blood Safety
**Countries with respective institution responding to the brief survey (n = 9)**	
Austria	Austrian Federal Ministry of Health, Vaccines Department
Bulgaria	Ministry of Health, National Centre of Infectious and Parasitic Diseases
Croatia	Croatian Institute of Public Health, Immunisation Department
Cyprus	Cyprus Ministry of Health, Directorate of Medical and Public Health Services
Czech Republic	National Institute of Public Health, Department of Infectious Disease Epidemiology
Estonia	Public Health Administration, Health Protection Inspectorate
France	French National Public Health Agency, Institute for Public Health Surveillance
Greece	Hellenic Centre for Disease Control and Prevention, Department for Surveillance and Intervention
Slovenia	National Institute of public Health, Centre for Communicable Diseases
**Countries with respective institution responding to the basic set of five questions after the survey deadline (n = 2)**	
Luxembourg	Ministry of health, Directorate of Health
Slovakia	Public Health Authority, Department of Epidemiology

IIS: Immunisation Information Systems; ECDC: European Centre for Disease Control and Prevention; EU/EEA: European Union/European Economic Area.

### Governance and financial support

Among the 27 countries who responded to either the comprehensive or brief survey or the basic set of five questions, 17 provided information on governance and financial support. In the survey, governance was defined as ‘the body at national or regional level that is in charge of the day-to-day management of the IIS and of the data contained in the system’.

For eight of the 13 countries with national systems, governance of the IIS is the sole responsibility of the National Institute of Public Health (NIPH). For two countries governance is held by the Ministry of Health (MoH), for Latvia it is held by the National Health Service (NHS), in Romania it is held by both the NIPH and MoH, and in Slovakia it is held by the National Health Information System (NHIS). 

Among the four countries with subnational systems, i.e. Belgium (described through Flanders), Spain (described through Andalucía) and the United Kingdom (UK) (described through England), governance is held by subnational or regional health authorities. In Portugal (described through mainland) it is held by both the NIPH and MoH (Table 2).

**Table 2:** Overall descriptions of the IIS in countries providing information on governance in ECDC surveys, EU/EEA, 2016 (n = 17 countries)

Country	Name of the IIS	Year established	National (N)/subnational (S)	IIS governance	Financial resources	IIS meets US CDC definition [1]
Belgium (Flanders)	Vaccinnet	2005	S	RHA	RG	Yes
Denmark	The Danish Vaccination Register (DDV)	2013	N	NIPH	NG	Yes
Finland	The National Vaccination Registry	2011	N	NIPH	NG	Yes
Germany	‘KV-Impfsurveillance’ [‘Associations of Statutory Health Insurance Physicians (ASHIP) vaccination monitoring’]	2011	N	NIPH	NG	No
Hungary	Országos Szakmai Információs Rendszer (OSZIR) Védőoltási és oltóanyag logisztikai alrendszer	2014 piloting	N	NIPH	NG	Yes
Iceland	Central Immunisation Register	2007	N	NIPH	NG	Yes
Ireland	School Immunisation System (SIS)	2011	N	MoH	NG	Yes
Latvia	National e-Health System	2016 piloting	N	NHS	NG and EU funds	Yes
Malta	National Immunisation Electronic Database	2009	N	MoH and Primary Healthcare	NG	Yes
Netherlands	Praeventis	2005	N	NIPH	NG	Yes
Norway	SYSVAK – Norwegian Immunisation Registry	1995	N	NIPH	NG	Yes
Portugal (mainland)	Vacinas	2003 (2017)^a^	S	NIPH and MoH	NG	Yes
Romania	National Electronic Registry of Immunization	2011	N	NIPH and MoH	NG	Yes
Slovakia	National Health Information System	Unknown, piloting	N	NHIC	NG and EU funds	NA
Spain (Andalucía)	Módulo de vacunas DIRAYA	2016	S	RHA	RG	Yes
Sweden	National Vaccination Registry	2013	N	NIPH	NIPH	No
United Kingdom (England)	Child Health Information System	Late 1980s	S	RHA	NG	No^b^

ECDC: European Centre for Disease Control and Prevention; IIS: Immunisation Information System; EU/EEA: European Union/European Economic Area; MoH: Ministry of Health; NA: not applicable; NG: national government; NIPH: National Institute of Public Health; NHIC National Health Information Centre; NHS: National Health Service (subordinate to MoH); RG: regional government; RHA: regional health authority; US CDC: United States Centers for Disease Control and Prevention.

^a^ An IIS is in place in mainland Portugal since 2003 (SINUS). A new system, Vacinas, is being piloted that will include additional features to the SINUS system.

^b^ Some subnational systems in the United Kingdom (England) fit the US CDC definition while others do not.

Financial support for the IIS comes from the national government for thirteen countries. In Latvia and Slovakia the IIS is funded by the national government and EU funds. The regional government finances the IIS in Belgium (Flanders) and Spain (Andalucía).

### Implementation status of Immunisation Information Systems 

The status of implementation of IIS in the 27 countries is as follows (Figure). 

**Figure:**
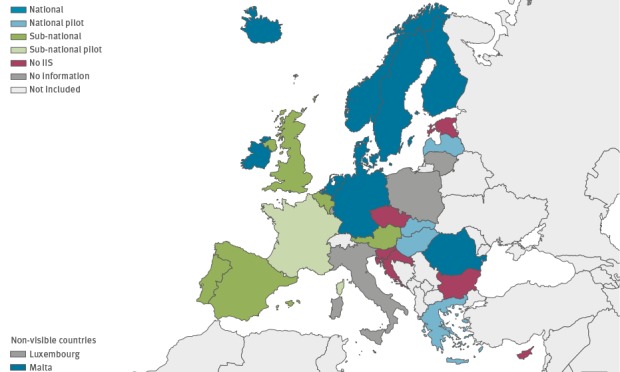
Status of implementation of Immunisation Information Systems in EU/EEA countries, 2016 (n = 27)

EU/EEA: European Union/European Economic Area.

Germany and Sweden have national systems that do not have the ability to consolidate immunisation histories for use at point of clinical care. Their systems only provide aggregated data on vaccinations at population level.

#### 

##### Countries with Immunisation Information Systems in place

Eight countries have a currently operating national system that meets the US CDC definition of an IIS, i.e. Denmark, Finland, Iceland, Ireland, Malta, the Netherlands, Norway and Romania. In Finland the IIS includes more features than specified in the US CDC definition.

Two countries (Germany and Sweden) have national systems in place that do not fully meet the US CDC definition of an IIS. In particular, their systems have no ability to consolidate immunisation histories for use at point of clinical care and only provide aggregated data on vaccinations at population level. 

Five countries have more than one subnational IIS, including Austria (number not specified), Belgium (Flanders, with the system also covering parts of Brussels, and in the Walloon region where the system also covers parts of Brussels), Portugal (mainland and Madeira), Spain (Andalucía, Illes Balears, Cataluña, Comunidad Valenciana, Castilla y León, Galicia, Comunidad de Madrid and Región de Murcia) and the UK (England, Northern Ireland, Scotland and Wales). For Belgium, Portugal, Spain and the UK, the survey describes the systems in operation in Flanders, mainland Portugal, Andalucía and England respectively. The systems in Belgium, Portugal and Spain fulfil the criteria of the US CDC IIS definition. The UK systems vary, some systems do meet the CDC definition of an IIS while others do not. This information was not available for Austria as they completed the short version of the survey where this question was not asked.

##### Countries piloting Immunisation Information Systems

Four countries, Greece, Hungary, Latvia and Slovakia are piloting a national system. Latvia had planned to pilot its system in 2017. 

France is piloting more than one subnational IIS. Bulgaria is piloting one subnational IIS. Among the countries piloting an IIS, whether at sub-national or national level, how the IIS was defined was only provided by Hungary and Latvia, as these two countries participated in the comprehensive survey. Both countries had an IIS fitting the US CDC IIS definition.

##### Countries with no Immunisation Information Systems

Six countries, including Croatia, Cyprus, Czech Republic, Estonia, Luxembourg and Slovenia have no IIS in operation or being piloted. Cyprus, Estonia, Luxembourg and Slovenia all have concrete plans to implement an IIS in the future.

### Characteristics of Immunisation Information Systems 

The results discussed in the following sections are based on questions only included in the comprehensive survey, hence only the 16 countries that responded to this survey (Table 1) are included in the sections below.

#### 

##### Immunisation Information Systems definition

Of 16 countries, which participated in the comprehensive survey, 13 have systems fitting the US CDC definition of an IIS [1] (Box and Table 2). In Finland the definition exceeds the US CDC definition in that the system is also used at individual level to provide immunisation information for use in surveillance, vaccine efficacy and impact studies. 

IIS in two countries (Germany and Sweden) do not fulfil the criteria of the US CDC definition of an IIS. The subnational systems in the UK (England) are varied, with some fulfilling the US CDC definition and others not. In Germany the system is based on insurance claims data from all physicians providing medical services (including vaccinations) to the statutory health ensured population in Germany (around 85% of the total population). Physicians or vaccination providers (at the point of clinical care) do not have access to this database. In Sweden the objective of the national vaccination register is to improve monitoring of the national vaccination programmes and is not used by vaccination providers in determining appropriate client vaccinations at the point of clinical care. In the UK (England) availability of vaccination history at point of clinical care is variable. In primary care, it is dependent on the supplier of the General Practice Information Technology (GP IT) system and the local Child Health Information System while in secondary care it is not available.

##### Immunisation Information Systems software

In 15 of the 16 countries, the government authority is the owner of the IIS software; whereas in the UK (England), there are five major private sector software suppliers. Fifteen of 16 countries provided information on software source code development, this information was missing for Hungary. Seven countries used a private company and six countries used programmers from the government authority. Two countries systems were developed by a mix of private and government programmers. 

Fourteen of the 16 countries provided information on the type of software used. Seven countries used commercial software. Three countries, Germany, Latvia and Spain (Andalucía) had both a partially open and partially commercial source. In Finland and Malta, it was open source with no license required, whereas in Romania, it was a free to use software, but a license was necessary. In Portugal (mainland), the software was developed specifically by the MoH. Information on the type of software was missing for Belgium and Hungary.

The survey did not collect elements related to data hosting, applied standards and system architecture.

##### Core attributes

Information included in the IIS is fed by a population registry in 13 of 16 countries. Of these 13, seven used the civil population registry, three used the healthcare registry, Denmark and Iceland used both the civil and the healthcare registries and Finland’s system is fed by patient data system records. In Germany, Ireland and Romania personal data are entered manually when the patient comes for their first vaccinations. Ten of 16 countries reported that an individual vaccination record was created automatically in the IIS database when a live birth is registered (or a time later). In seven countries vaccination records were also set-up automatically at the time of immigration to the country. 

Ten countries record life-long vaccination data in the IIS with no restriction of age or vaccination setting (Table 3). The IIS in Ireland records only vaccinations in the recommended school-based vaccination programme. Hungary, the Netherlands, Romania, Sweden and the UK (England) do not include >18 year-olds vaccination data in their systems.

**Table 3:** Population included, recording of individuals and vaccinations in the IIS of EU/EEA countries, 2016 (n = 16 countries)

Country	Does the IIS record whole-of-life vaccination data?	Each immunised individual is recorded with a unique UI?	Does the IIS use the UI given to citizens at birth or immigration?	Can vaccinations administered in the past be recorded?	Can vaccinations administered abroad be recorded?	Are vaccination data entered selected from a list?
Belgium (Flanders)	Yes	Yes	Yes	Yes	Yes	Yes
Denmark	Yes	Yes	Yes	Yes	Yes	Yes
Finland	Yes	Yes	Yes	Yes	Yes	Yes
Germany	Yes	Yes	**No**	**No**	**No**	Yes
Hungary	**No**	Yes	**No**	Yes	Yes	**No**
Iceland	Yes	Yes	Yes	Yes	Yes	Yes
Ireland	**No**	Yes	**No**	Yes	**No**	Yes
Latvia	Yes	Yes	Yes	Yes	Yes	Yes
Malta	Yes	Yes	Yes	Yes	Yes	Yes
Netherlands	**No**	Yes	Yes	Yes	Yes	Yes
Norway	Yes	Yes	Yes	Yes	Yes	Yes
Portugal (mainland)	Yes	Yes	**No**	Yes	Yes	Yes
Romania	**No**	Yes	**No**	Yes	Yes	Yes
Spain (Andalucía**)**	Yes	Yes	Yes	Yes	Yes	Yes
Sweden	**No**	Yes	Yes	**No**	**No**	Yes
United Kingdom (England)	**No**	Yes	Yes	Yes	Yes	Yes

IIS: Immunisation Information Systems; UI: unique identifier.

All 16 systems use a unique personal identifier for each immunised individual recorded in the IIS (Table 3). In 11 countries the unique identifier used in the IIS is the same one that is given to citizens at birth or immigration. In Portugal (mainland) the unique identifier is the one given for healthcare services, whereas for four countries the unique identifier is specific to the IIS.

Fourteen of 16 countries can record vaccinations administered in the past and 13 systems can record vaccinations administered abroad (Table 3). This is not possible in Ireland, Germany and Sweden. In four countries with subnational systems (Belgium (Flanders), Portugal (mainland), Spain (Andalucía) and the UK (England)), vaccinations administered in other regions can be recorded in the IIS. The ability of the various systems in the EU to automatically share data was not assessed as it is known to not occur in the EU.

For 15 countries, to ensure that a vaccination entry is valid, vaccine providers are able to select the vaccination to be administered from a list included in the system. For seven countries the data captured in the IIS is validated automatically by the system through pre-set rules and similar. The measures that countries use to audit the quality of the data in the IIS was not captured in this survey.

When a vaccine is administered, vaccination information is entered into the IIS in real-time in Denmark, Iceland, Malta, Norway, Portugal (mainland), Spain (Andalucía), Sweden and the UK (England).

##### Use for surveillance purposes

In order to estimate vaccination coverage nine countries of 15 use the civil population registry as the source of denominator data for the IIS. Germany, Hungary, Portugal (mainland) and Spain (Andalucía) use healthcare registries as the denominator. In Ireland the number is manually obtained from the school census and Romania uses the number of newborn children from maternity hospitals. Information on vaccine coverage denominator was missing for Latvia.

In order to compute aggregated vaccination uptake on the smallest administrative area, eight countries of 16 used nomenclature of territorial units for statistics (NUTS) 3 [22] and Hungary computed on NUTS 1. Seven countries were able to calculate coverage below NUTS 3: Sweden and Denmark could compute data at municipality level, Belgium (Flanders), Iceland and the Netherlands at postal code level, and Portugal (mainland) as well as Finland at healthcare centres’ level.

Six countries of 16 can use their systems to record adverse events following immunisation (AEFI). In Belgium (Flanders), AEFIs can be added and marked in colour, so at the time of future vaccination when the provider goes online this can clearly be seen. In two countries (Ireland and Latvia) the system is used for routine passive reporting of AEFIs to health authorities. In Portugal (mainland), the system allows recording of AEFIs, however reporting to fulfil regulatory requirements is done through another system.

Eleven countries can link their IIS with various health outcome registers. For five countries some of these registers are integrated within the IIS and for the other six countries linkage with other health outcome registers is either routinely carried out or performed for specific purposes. Thirteen of 14 countries allow public health organisations to use IIS data for research, such as in vaccine effectiveness studies and safety studies. Latvia has not yet defined this and there was no information from Spain (Andalucía) for this question. In five of these 14, other non-public health organisations can have access to the IIS data for research.

Ten countries can use their IIS to identify unvaccinated individuals in the event of an outbreak.

##### Use for management purposes

Five of 16 countries (Latvia, Malta, the Netherlands, Portugal (mainland) and the UK (England)), have automated systems that can send reminders to people who are due to get vaccinated. The systems in Latvia, Spain (Andalucía), Portugal (mainland) and the UK (England) can send automatic reminders to the vaccine provider to call a patient for the next vaccination.

In five of 15 countries (Denmark, Iceland, Latvia, Norway and Portugal (mainland)), the vaccine recipient or guardian has access to the IIS. There was no information available for Hungary for this question. These five countries, plus Belgium (Flanders), also provide vaccine recipients with the ability to independently obtain an individual immunisation history that is accepted as an official immunisation record directly through the IIS or through an exchange platform.

Regarding outputs from IIS systems, five of 16 countries have a system that allows vaccine providers to identify which vaccines to administer based on the recipient’s age, previous vaccination, allergies, travel and risk factors. In Belgium (Flanders), Portugal (mainland) and Spain (Andalucía), the IIS can be used to communicate information on new vaccines, updated policies, safety concerns and out-of-stock situations to vaccine providers. Thirteen countries can use it to identify individuals who are incompletely vaccinated according to age and ten countries can use it to record reasons for refusing vaccination.

##### Challenges in implementation

Countries had encountered a number of challenges during the different phases of IIS implementation.

The most common challenges faced during the decision to set up an IIS were a lack of human resources (12/15 – no answer from Spain (Andalucía) and a lack of funding (11/15 – no answer from Spain (Andalucía)), followed by issues relating to data protection (9/14 – no answer from UK (England) and Spain (Andalucía)).

During the design phase, challenges faced by most countries included defining the functions required by the system (12/15 – no answer from UK (England)) and a lack of standards to provide a point of reference for developing the system (10/15 – no answer from UK (England)), and defining the core dataset of information to be collected (10/15 – no answer from UK (England)).

During the early use phase (those countries that were piloting IIS were asked to leave this section blank), the main issues encountered included training vaccine providers to use the system (10/14 – Latvia piloting, no answer from UK (England)), validation of data entered by different users (9/13 – Latvia piloting, no answer from Malta and UK (England)) and quality control of data completeness (9/13 – Latvia piloting, no answer from Malta and UK (England)).

For the nine countries with no IIS in place or in the initial stages of implementation and who answered the brief survey, the main challenges were a lack of standards (7/8 – no answer from Austria), data protection issues (7/9) and issues relating to governance and ownership of the system (6/8 – no answer from Austria).

## Discussion

The findings of the survey provide information on the extent of IIS implementation and systems functionalities in 27 EU/EEA countries. Most EU/EEA countries either have an operational IIS or are piloting one. Of the countries who have no systems in operation, Estonia, Luxembourg and Slovenia all have concrete plans to implement an IIS as part of their larger eHealth strategies in the coming years and Cyprus plans to implement a system as part of the new National Health System [23]. This wide scale implementation of IIS is a major achievement and represents a substantial step towards improving the delivery and the monitoring of vaccination programmes in the EU/EEA as part of a broader strengthening of health service capacity.

Monitoring vaccination programmes relies not only on accurate and complete denominators and numerators for calculating vaccination coverage but also ensuring that the data captured in the system is reliable. The quality of data contained in each of the IIS in operation was not assessed through this survey. However, in regards to the source used for denominator data, an IIS that is populated automatically from birth and civil population registers, from national health insurance schemes or school registration is more likely to be complete. The countries who responded to the survey were advanced in this area. All countries used either the civil population registry, healthcare registries, school census or number of newborn children from maternity hospitals as data sources. All countries were also able to estimate coverage at subnational levels. In Finland and Portugal (mainland) for example, coverage can be assessed for populations with the same postal code and for populations using the same healthcare centre. At a population level, it is particularly important to be able to assess coverage in areas that are at high risk for low vaccination uptake. For example, in the Netherlands, the IIS can monitor coverage in areas of known low vaccination coverage, such as the ‘Bible Belt’ area, so as to adapt interventions [24].

For the numerator, the recording of vaccinations and vaccine details are also critical pieces of information required for coverage calculation. To minimise errors, manual data entry of vaccine details should be avoided. All the countries can validate the data entered into the IIS through methods such as bar code readers (e.g. in Spain (Andalucía)), drop-down menus to select from a pre-defined list of vaccines (in 15 countries), linking to a product database (e.g. in Finland and Hungary) or uploading from electronic medical records by web services (e.g. in Belgium (Flanders)). This is another major strength of the systems operating in the EU/EEA in that they do not rely on manual data entry to capture information on vaccinations received.

In regards to the characteristics of an IIS it is desired that the data captured in the IIS are complete, timely and of high quality. To ensure completeness, the IIS should ideally be populated with data from all vaccine delivery sites (whether public or private providers), they should cover the entire population and hold information on all vaccines recommended by health authorities regardless of funding. Many countries’ systems only capture vaccines provided in public health services and for those vaccines that are recommended and funded under the national immunisation schedule. To ensure timeliness and reduce underreporting it is essential that the time between vaccination and the information being entered into the IIS is minimised so that the information is in real-time. This is particularly relevant during emergency situations [25] or outbreaks when the prompt identification of unvaccinated people is necessary [26]. Systems in Belgium (Flanders), Denmark, Finland, Germany, Iceland, Latvia, Malta, Norway, Portugal (mainland) and Spain (Andalucía) allow for life-course vaccination information to be recorded. In 14 countries it is also possible to add vaccinations that were administered before the implementation of the IIS.

The IIS can also be used as a tool for informing public health decisions and research beyond vaccination coverage. The IIS constitutes large datasets that can be used in pharmaco-epidemiological studies to assess vaccine safety and effectiveness. Interoperability of the IIS with other health information systems has been used in studies such as the investigation of narcolepsy with pandemic influenza vaccination in Finland [27]; and similarly to investigate and provide reassurances following signals or claims of adverse effects, such as the investigation of the occurrence of adverse events affecting adolescents girls after human papillomavirus (HPV) vaccination in Sweden and Denmark [28]; the association of thimerosal-containing vaccines and autism in Denmark [29]; and the investigation of vaccines and auto-immune disorders in France [27].

Other important features of an IIS include automated reminder/recall, access and education. At present, systems in Latvia, Malta, the Netherlands, Portugal (mainland) and the UK (England) can send reminders to people who are due to get vaccinated and the systems in Latvia, Portugal (mainland), Spain (Andalucía) and the UK (England) can send automatic reminders to the vaccine provider to call a patient for the next vaccination. Providing public access to the IIS and allowing vaccine recipients to print immunisation records are valuable features. Vaccine recipients can view their records in the IIS in six countries (Denmark, Iceland, Latvia, Malta, Norway and Portugal (mainland)). Six countries allow recipients to directly access an official immunisation record through the IIS. By providing vaccine recipients with some level of ownership over their records and having online access to information on particular vaccines and the disease they protect against may be beneficial to the uptake of vaccination. Such systems also provide the opportunity for being used as educational tools for both vaccine providers and recipients. This can be done by including an easily accessible platform that provides clear information and visualisation of data, using, for example, dashboards. The systems in Denmark and Norway are linked to a web-based application that allows users to visualise in real-time the coverage at communal level with a graphical snapshot of current or historical vaccination coverage trends. This can be useful for informing interventions and raising community awareness.

The implementation of an IIS is a significant commitment at national and subnational levels in terms of financial investment to cover both human resources and technology developments as well as ensuring supportive legislation to allow for personal data to be recorded and used. Some of the challenges identified through the survey include the need for human resources and funding. Other challenges brought forward included the lack of standards. ECDC is well-placed to facilitate such exchange and collaboration in a more systematic way such as supporting EU countries in developing and agreeing to a minimal set of functionalities for an IIS, as a reference to help countries with IIS in the development phase. ECDC could also help in identifying lessons to be learned from other countries outside the EU/EEA. In the US, individuals and organisations with an interest in IIS have formed the American Immunization Register Association (AIRA), which in collaboration with the US CDC, has published platform neutral IIS best practices and standards [30]. Also the experience gathered from other countries outside of the EU with long-standing experience in IIS such as Australia and Canada will serve the EU setting. The Australian Immunisation Register was established in 1996 initially to record vaccinations given to Australian children up to seven years of age. In January 2016 the register was expanded to include vaccination history for adolescents up to the age of 20. It then further progressed later in 2016 to capture all vaccines given as part of the national immunisation programme given to people of all ages and thereby provides a whole of life immunisation history [31].

The survey had some limitations. First, the survey did not include interviews with immunisation programme managers or other key stakeholders, such as decision-makers, programme and IT staff, which would have been useful to provide a more detailed overall picture of the IIS in countries surveyed. Second, the survey did not cover the transition period from paper-based to electronic registries. Last, the survey did not cover in detail the measures that countries use to audit the quality of the data in the IIS, such as the use of a paper-based questionnaire to compare with the data captured in the IIS. Despite these limitations this survey has provided critical information about systems across the EU/EEA and can be used as a further step for in-depth assessment of system performance. The survey also provided key information about the challenges and barriers that countries faced at different stages of implementation of the IIS. Sharing this knowledge and lessons learnt can potentially assist countries to overcome these issues especially those countries that are in the early stages of developing/using an IIS or are planning to implement a system in the future.

### Conclusions

Within the EU/EEA, countries vary considerably with respect to recommended vaccines, organisation of health services, mandate of public health agencies, legislation on confidentiality and other relevant factors. Despite this, the exchange of information and experience between national programmes has been useful in the development of IIS in many EU/EEA countries.

The setting up of an IIS is an important commitment for countries and requires careful planning of resources and time. ECDC can play an important role in bringing together key stakeholders, defining common areas of work and challenges, and facilitating exchange of knowledge and experience in order to support countries to implement or upgrade an IIS. The current focus on eHealth in the EU and at national level provides the perfect opportunity for IIS to become an integral part of electronic health systems.
